# Supplementary material for: Stereotactic Body Radiotherapy as a Salvage Therapy after Incomplete Radiofrequency Ablation for Hepatocellular Carcinoma: A Retrospective Propensity Score Matching Study
Source: Cancers (Basel). 2019 Aug 5;11(8):1116. doi: 10.3390/cancers11081116 (PMC6721575; doi:10.3390/cancers11081116)
Supplement: Supplementary file 1 [file cancers-11-01116-s001.zip › Supplementary Table S1.docx]

| Supplementary Table S1. Patient and Residual Tumor Characteristics Excluded from Propensity Score Match (PSM) | | | | | | |
| --- | --- | --- | --- | --- | --- | --- |
|  |  |  | | | | |
|  |  | RFA group (n = 16) | SBRT group (n = 10) | | *P* value | |
| Sex, M/F |  | 16/0 | 10/0 | |  | |
| Age, years |  | 54.50 (46.80-61.00) | 61.50 (58.20-68.20) | | 0.137 | |
| Cirrhosis, Y/N |  | 9/7 | 1/5 | | 1.000 | |
| B virus hepatitis, Y/N |  | 12/4 | 6/4 | | 0.712 | |
| Child-Pugh score, n | A5 | 16 | 9 | | 0.809 | |
|  | A6 | 0 | 1 | |  | |
|  | B7 | 0 | 0 | |  | |
| White blood cell, *10^9^/L |  | 4.70 (4.25-5.50) | 5.25 (4.46-5.96) | | 0.756 | |
| Platelet, *10^9^/L |  | 103.00 (87.50-118.00) | 155.00 (132.00-206.00) | | 0.012 | |
| Alanine aminotransferase, U/L |  | 39.70 (32.30-54.80) | 28.40 (21.00-32.60) | | 0.264 | |
| Aspartate aminotransferase, U/L |  | 32.30 (28.10-44.50) | 26.60 (21.40-35.50) | | 0.978 | |
| Alpha fetoprotein, ng/mL |  | 32.80 (4.74-1380.00) | 130.00 (3.91-842.00) | | 0.884 | |
| Tumor Size, cm |  | 1.60 (1.48-2.00) | 5.10 (4.70-6.80) | | <0.001 | |
| Location, peripheral/central |  | 5/11 | 2/8 | | 0.861 | |
| HCC adjacent to or invading main vessel, Y/N |  | 4/12 | 9/1 | | 0.005 | |
| HCC abutting the capsule, Y/N |  | 8/8 | 1/9 | | 0.096 | |
| Follow-up time, months |  | 37.00 (23.00-92.90) | 22.10 (11.20-28.70) | | 0.063 | |
| NOTE. Data presented as mean (interquartile range), unless otherwise noted. | | | |  | |  |
| Abbreviations: RFA, radiofrequency ablation; SBRT, stereotactic body radiation therapy; HCC, hepatocellular carcinoma. | | | | | | |
